# Defining the Akt1 interactome and its role in regulating the cell cycle

**DOI:** 10.1038/s41598-018-19689-0

**Published:** 2018-01-22

**Authors:** Shweta Duggal, Noor Jailkhani, Mukul Kumar Midha, Namita Agrawal, Kanury V. S. Rao, Ajay Kumar

**Affiliations:** 1 Drug Discovery Research Center (DDRC), Translational Health Science and Technology Institute (THSTI), NCR Biotech Science Cluster, Faridabad, 121001 India; 2 International Centre for Genetic Engineering and Biotechnology (ICGEB), New Delhi, 110067 India; 3 Department of Zoology, University of Delhi, Delhi, 110007 India

## Abstract

Cell growth and proliferation are two diverse processes yet always linked. Akt1, a serine/threonine kinase, is a multi-functional protein implicated in regulation of cell growth, survival and proliferation. Though it has a role in G1/S progression, the manner by which Akt1 controls cell cycle and blends cell growth with proliferation is not well explored. In this study, we characterize the Akt1 interactome as the cell cycle progresses from G0 to G1/S and G2 phase. For this, Akt1-overexpressing HEK293 cells were subjected to AP-MS. To distinguish between individual cell cycle stages, cells were cultured in the light, medium and heavy labelled SILAC media. We obtained 213 interacting partners of Akt1 from these studies. GO classification revealed that a significant number of proteins fall into functional classes related to cell growth or cell cycle processes. Of these, 32 proteins showed varying association with Akt1 in different cell cycle stages. Further analyses uncovered a subset of proteins showing counteracting effects so as to tune stage-specific progression through the cycle. Thus, our study provides some novel perspectives on Akt1-mediated regulation of the cell cycle and offers the framework for a detailed resolution of the downstream cellular mechanisms that are mediated by this kinase.

## Introduction

The mammalian cell cycle consists of an ordered series of events and is a highly coordinated and regulated process^1^. Cell cycle requires the activation of many stage specific signalling molecules as well as that of regulatory cell cycle proteins. Proliferation of cells depends on progression through four distinct phases of the cell cycle-G0/G1, S, G2 and M, which are regulated by various proteins interacting in signalling pathways in complexes^2^. The dynamic constitution of protein-protein interactions in signalling pathways is important to coordinate cellular functions in response to extrinsic or intrinsic proliferation signals^3,4^.

Cell growth, a process that coordinates with cell cycle during cell doubling, is defined as an increase in cell mass and size^5^. This leads to lower surface area to volume ratio in cells and spurs cells to divide. A key regulator of cell growth is Akt (also known as protein kinase B or PKB), a serine/threonine kinase that also regulates other cellular functions like proliferation, glucose metabolism, and survival^6,7^. In humans, there are three Akt genes-Akt1 (PKBα), Akt2 (PKBβ), and Akt3 (PKBγ), which share a high degree of amino acid sequence similarity and are believed to have similar specificity for their primary substrates^8^. However, their functional spectrum shows variety and some redundancy too. Akt1 has a suggested role in cell proliferation and survival, while Akt2 exercises its control over metabolism and Akt3 which is more dominant in brain tissues is implicated in mediating cell growth processes along with Akt1^9,10^.

Akt1 is involved in the regulation of cell proliferation and transformation. The wide variety of targets available for Akt1 allows it to stimulate cellular proliferation through myriad downstream substrates with multiple implications on cell-cycle progression and regulation^6,11,12^. When mitogenic stimulation is provided to mammalian cells in quiescent (G0) stage, a rapid trigger in a number of biochemical signalling cascades is observed. One among such cascades is the PI3K/Akt pathway, which serves to promote cell growth via activation of two key enzymes, mTOR and p70S6K^13,14^. Growth factor mediated Akt1 activation also leads to release of the cells from G0 phase and commits them into the cycle by driving them into the G1 phase. This in turn ensures the crossover of G1/S checkpoint for their entry into the synthesis phase. Yun *et al*. recently demonstrated that Akt1 was also crucial for G1/S transition^15^. However, precise mechanism by which Akt1 regulates the cell cycle, and also the manner in which it coordinates cell growth and proliferation, remains unclear. Here it seems possible that a resolution of the protein-protein interactions that Akt1 engages in, and an understanding of how such interactions are modulated as cells progress through the cycle, will shed some light on this question. This understanding is clearly relevant given that Akt1 is overexpressed in majority of the cancers^10^.

Our focus in the present study therefore, was to characterize the Akt1 interactome, and also to define any alterations in its composition that accompanied progression of cells through individual stages of the cell cycle. For this we employed Akt1-overexpressing HEK293 cells, which were subjected to affinity purification coupled with mass spectrometry (AP-MS). Further, to resolve between the individual cell cycle stages, we used the technique of selective isotope labelling of amino acids in cell culture (SILAC). These studies identified 213 proteins to interact either directly or indirectly with Akt1. Of these, association of 32 varied with the cell cycle stage. A gene ontology (GO) classification of the Akt1 interactome revealed that a significant number of the component proteins were derived from functional classes that related either to cell growth, or to cell cycle processes. Subsequent experiments revealed that at least a significant proportion of the proteins that associated with Akt1 in a dynamic manner were indeed involved in influencing progression of cells through the cycle. Interestingly, these proteins appeared to exert counteracting effects so as to tune stage-specific progression and, thereby, the overall population doubling time (PDT) of the cells. Thus, in addition to shedding some novel perspectives on Akt1-mediated regulation of the cell cycle, our studies also provide the framework for a detailed resolution of the downstream cellular mechanisms that are mediated by this kinase.

## Results

### Akt1 overexpression and characterization in HEK293 cells

**Figure 1:**
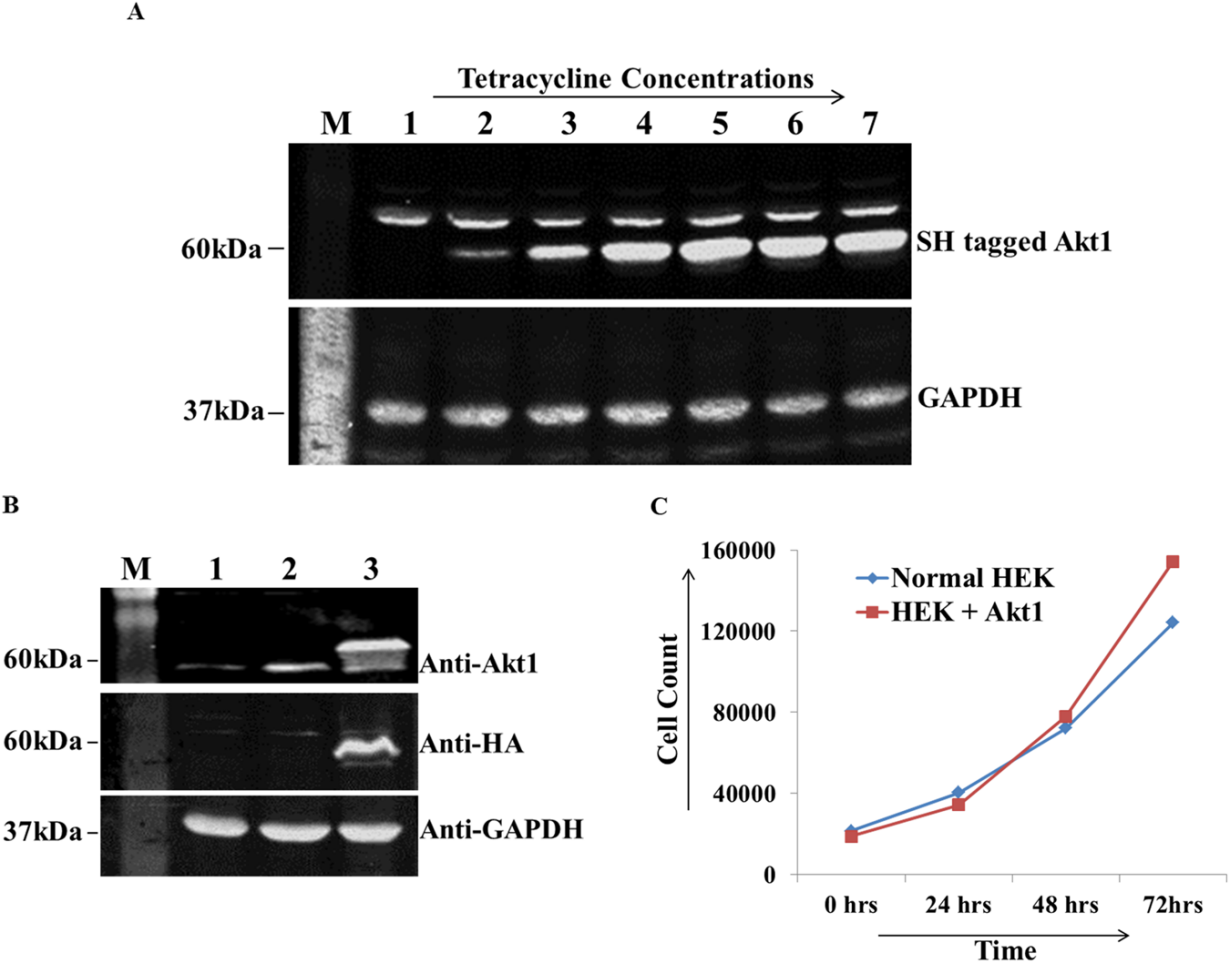
Standardization of Akt1 over-expression (**A**) and subsequent confirmation of expression by western blotting (**B** and **C**). (**A**) Standardization of Akt1 expression over a range of Tet concentrations (0.1–5.0 µg/ml); Lane M: Marker; Lane 1: Uninduced sample; Lanes 2–7 depict various Tet concentrations used-Lane 2: 0.1 µg/ml; Lane 3: 0.25 µg/ml; Lane 4: 0.5 µg/ml; Lane 5: 1.0 µg/ml; Lane 6: 2.5 µg/ml; Lane 7: 5.0 µg/ml. (**B**) Normal and Akt1 expressing HEK293 cells (in presence and absence of Tet) probed for Akt1 and HA probe, simultaneously; GAPDH was used as a loading control. Lane M: Marker; Lane 1: Lysate from normal HEK293 (minus Tet); Lane 2: Lysate from Akt1 expressing HEK293 (minus Tet); Lane 3: Lysate from Akt1 expressing HEK293 (+Tet). (**C**) Effect of Akt1 transfection on population doubling time.

In this study, gateway compatible Akt1 entry vector was used to generate an expression construct which was later co-transfected with pOG44 recombinase to generate a Tet- inducible Akt1 expressing cell line (Methods). Optimal Akt1 expression was observed at 1 µg/ml Tet concentration (Fig. 1A) and therefore this Tet concentration was used to induce Akt1 expression in all experiments. Significant increase in Akt1 expression was seen in Tet induced Akt1 expressing cells as compared to normal and un-induced cells (Fig. 1B). Similarly, when probed for HA tag, only induced transfected cells gave a positive signal with anti-HA antibody as shown in Fig. 1B.

Next, the effect of Akt1 transfection on PDT of HEK293 cells was determined by trypan blue staining method (Methods). Akt1-overexpressing cells showed significant decrease in PDT (by 4.92 hours) as compared to normal cells, which had a doubling time of 28.53 hours (Fig. 1C). These results confirm a functional role for Akt1 in driving HEK293 cells through cell cycle.

Since our interest was to probe the role of Akt1 in cell cycle regulation, we first optimized conditions for arresting cells in individual stages of the cell cycle. Normal and Akt1-overexpressing cells were arrested in respective cell cycle stages and analyzed by FACS following propidium iodide staining (Methods). As compared to untreated cells, G0/G1 population increased by 4.74% subsequent to overnight serum starvation and by 11.46% after aphidicolin treatment. Concurrently, a decrease by 3.5% and an additional decrease by 9.12% in G2/M populations were documented upon serum starvation and aphidicolin treatment, respectively. Since the histograms from FACS analysis present G0 and G1 population pooled as a single peak, the consequence of methods employed to arrest cells in respective phases could only be indicated by shift or change in intensity of G0/G1 peak. Likewise, in cells treated with nocodazole, G2/M population increased significantly by 36.11% while G0/G1 population depleted by 52.18%. Experimental incubation times and working concentrations of aphidicolin and nocodazole were determined from reports published elsewhere^16,17^.

**Figure 2:**
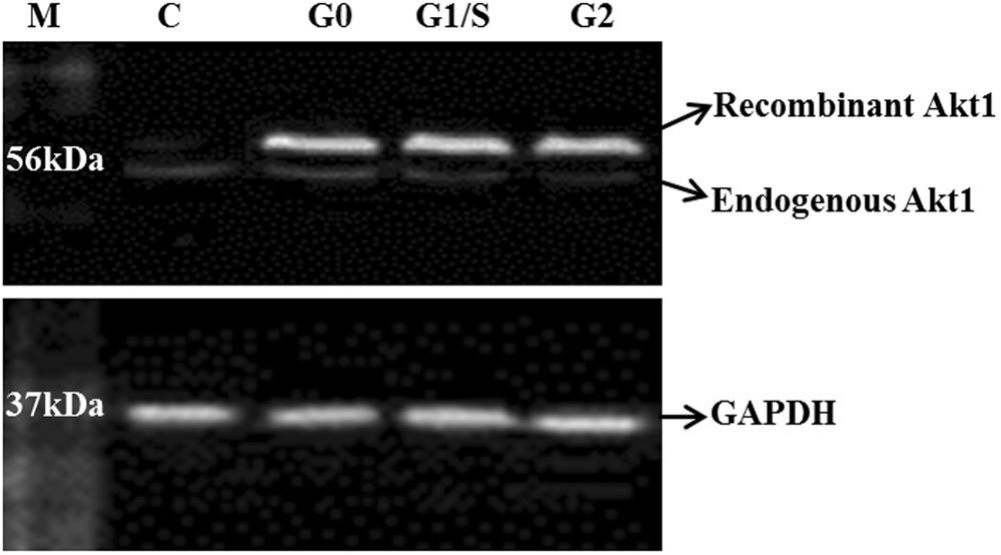
Cell lysate from un-induced and tetracycline induced HEK293 TREx cells arrested in different stages of cell cycle were probed with anti-Akt1 antibody. GAPDH was probed as loading control. Lane M represents protein molecular weight marker; Lane C represents Akt1 transfected but un-induced (minus tetracycline) HEK293 TREx cell lysate; Lanes G0, G1/S and G2 represent cell lysates from tetracycline induced Akt1 transfected HEK293 TREx cells arrested in G0, in G1/S transition and in G2 phase of the cell cycle respectively.

In order to check if Akt1 protein expression changes as the cell cycle progresses, cell lysates from Akt1-overexpressing cells arrested in different cell cycle stages were investigated by western blot, using GAPDH as the loading control (Fig. 2). Akt1 protein expression was found to be comparable across stages of the cell cycle (Fig. 2) when probed with Akt1 specific anti Akt1 antibody.

### The Akt1 interactome

**Figure 3:**
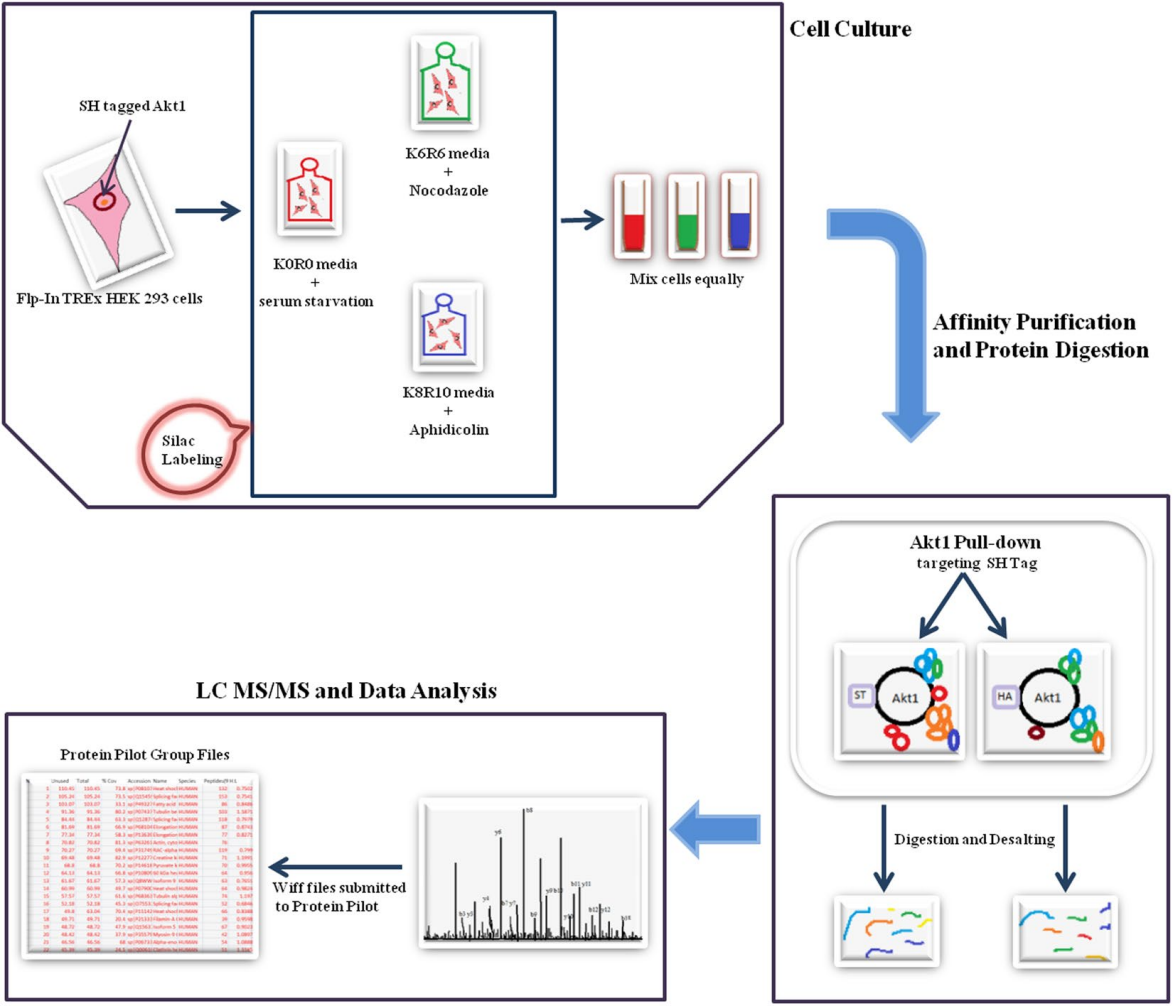
Depiction of workflow integral to delineating Akt1 interactome during the course of cell cycle progression by immunoprecipitation by targeting SH tag followed by LC-MS/MS mass spectrometry.

One of the primary aims of our study was to delineate the Akt1 interactome and to study how its composition alters during the course of cell cycle progression. It was anticipated that such an analysis would provide new insights into pathways through which Akt1 coordinates cell growth with the cell cycle, to drive proliferation. To achieve this, we employed the SILAC approach coupled with LC-MS/MS mass spectrometry. Broad outline of the workflow is depicted as a schematic diagram in Fig. 3.

The immuno-precipitated Akt1 interactors were analyzed in three separate experiments and proteins identified in all the three experiments at 1% G-FDR-fit were considered as putative interactors. This gave us a list of 252 Akt1 interactors. A representative chromatogram, depicting estimated FDR for Lys C and Trypsin digested G0 phase, G2 phase and G1/S phase samples, is provided in Supplementary Figure 1. This list was further pruned by excluding probable non-specific interactors. The latter were identified from parallel SH-tagged GFP expression in HEK293 cells and pull-down experiments (Methods Section). A total of 59 proteins were found as GFP interactors at 1% G-FDR-fit (Supplementary Table 1). Of these, 39 proteins overlapped with Akt1 interactome (Supplementary Table 2) and were removed as non-specific interactors from Akt1 interactor list.

**Figure 4:**
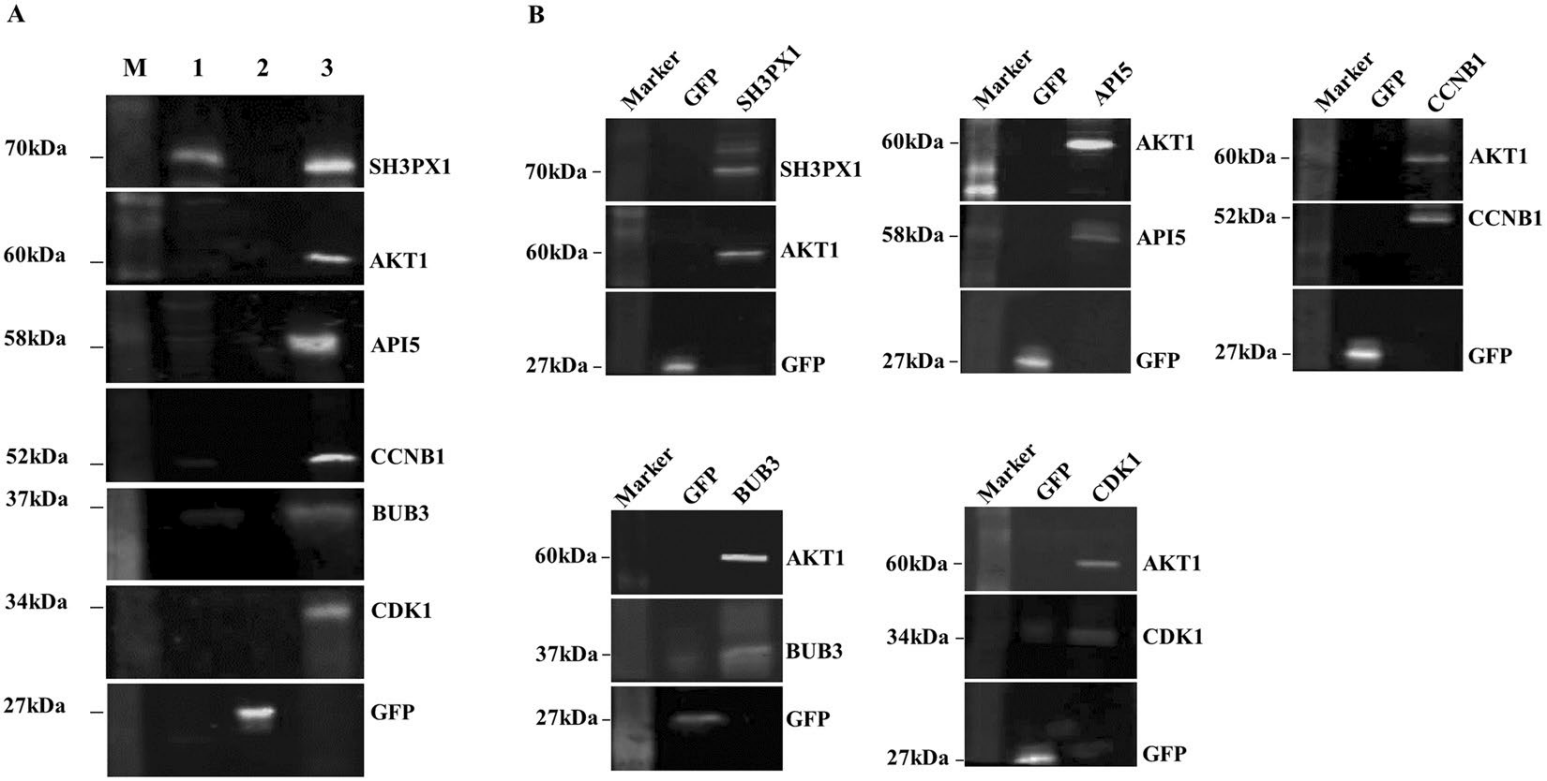
Co-IP and reverse Co-IP results to validate association between Akt1 and its interacting partners (**A**) Panel of western blots depicting results of Co-IP of API5, SH3PX1, CCNB1, BUB3 and CDK1 in Akt1 pull-down samples. Lane M: Marker; Lane 1: Lysate from normal HEK293; Lane 2: GFP Elution; Lane 3: AKT1 Elution. (**B**) Reverse- Co-IP results depicting successful detection of Akt1 protein in pull-down samples from API5, SH3PX1, CCNB1, BUB3 and CDK1; GFP was used as a negative control for both Co-IP as well as reverse Co-IP experiments.

After subtracting the non-specific interactors, a total of 213 unique and high-confidence interactors of Akt1 were obtained. A summary of information on the Akt1 interacting proteins identified in replicate sets and their quantitation estimates is tabulated as Supplementary Table 3 and their corresponding processed file depicting mean heavy/light or medium/light ratios for all 213 high-confidence interactors is provided in Supplementary Table 4. To validate the interactions observed, we immuno-precipitated Akt1 from HEK293 cells and probed it for the presence of five randomly selected proteins by western blot. Antibodies against API5, SH3PX1, CCNB1, BUB3 and CDK1 were used here and, consistent with our findings from mass spectrometry, the presence of all five proteins could be confirmed in the Akt1 immuno-precipitates (Fig. 4A).

Reverse co-immunoprecipitations further confirmed our findings. Immunoprecipitation from HEK293 cell lysates with antibodies against each of the above five proteins revealed the presence of Akt1, as detected by western blot, in all cases (Fig. 4B). Anti-Akt1 antibody was used here to probe for Akt1 as an interactor.

**Figure 5:**
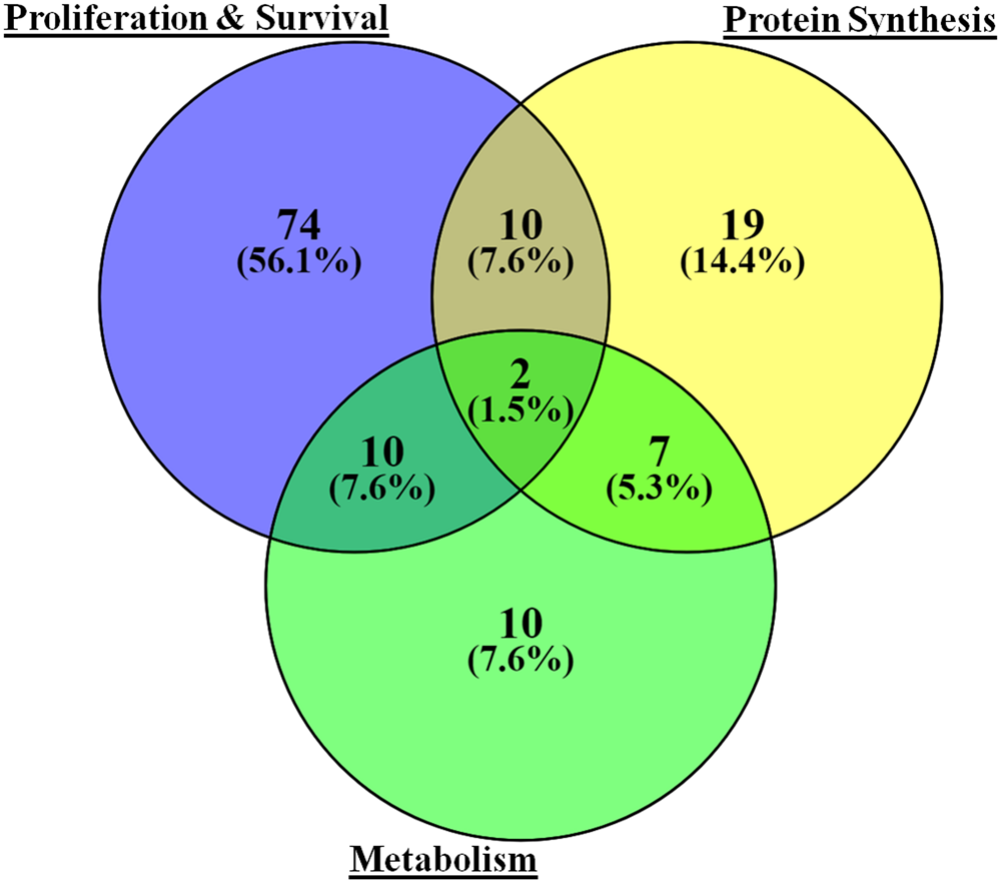
Venn diagram depicting GO distribution of Akt1 interactome into three major GO classes- Proliferation and Survival, Protein Synthesis and Metabolism.

We next performed a GO based annotation for all 213 Akt1 interactors using UniProt and EnrichR databases. Interestingly, 132 of these proteins derived from the functional classes proliferation and survival (n = 96), protein synthesis (n = 38), or metabolism (n = 29) (Fig. 5). While the former functional class relates to regulation of the cell cycle, the latter two classes can be directly implicated in processes contributing to cell growth. That is, these groups of proteins together likely mediate the mitogenic activity of Akt1. The remaining proteins broadly represented functions related to transport and maintenance of homeostasis, and were grouped under other GO classes (Supplementary Table 5).

### Modulations in the Akt1 interactome as a function of cell cycle progression

**Figure 6:**
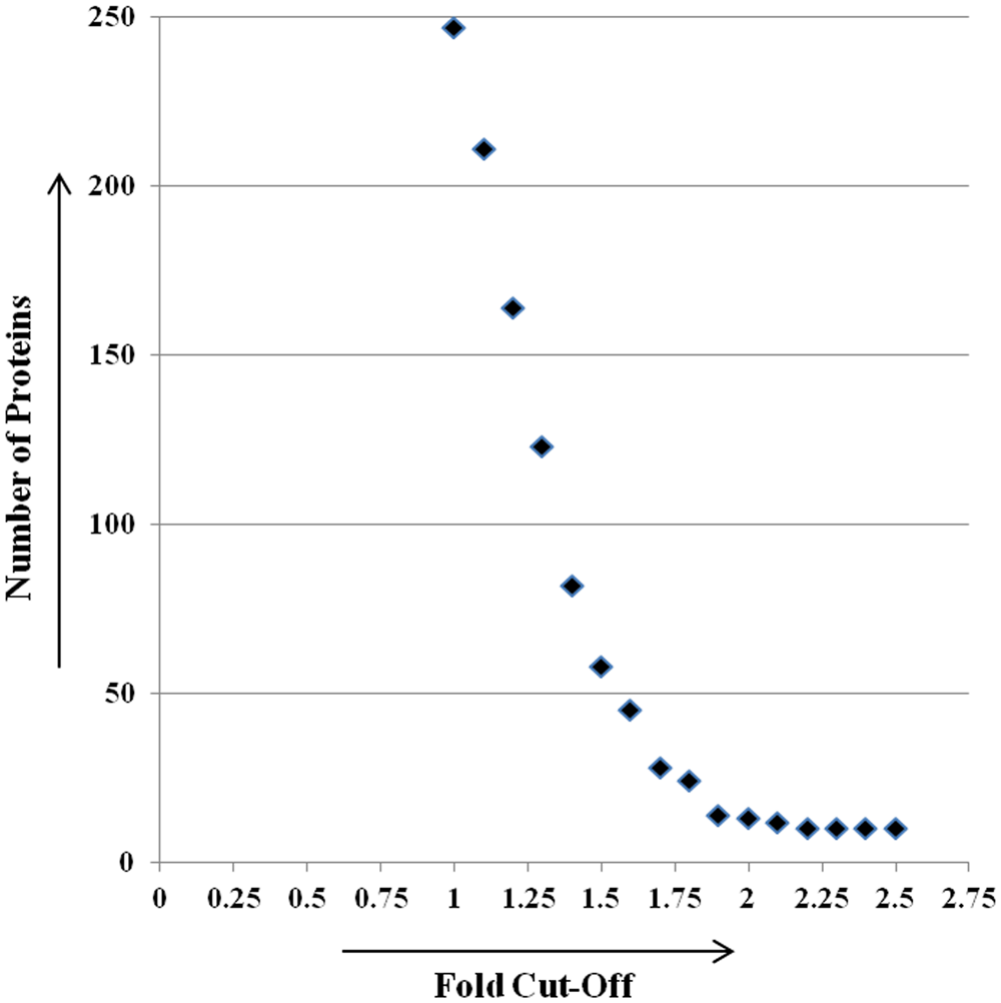
Scatter-plot depicting distribution of fold-change in association of interactors with Akt1 protein to determine threshold of significance. All interactors were grouped into 16 groups based on varying fold change from 1 to 2.5.

Alterations in composition of the Akt1 interactome, across individual cell cycle stages could be directly ascertained by determining ratios of the light, medium and heavy SILAC labels in peptides derived from the individual proteins in our mass spectrometric analysis. Ratios of heavy (G1/S) and medium (G2) SILAC labels over the light (G0) labelled counterparts revealed whether the extent of association of any given protein with Akt1 was altered either in the G1/S or G2 phases, relative to that in the G0 phase. Broadly, fold variation in SILAC ratios varied from 1 to 2.5-fold. To determine the threshold ratio that would be indicative of a significant change, we plotted the fold-change in association against number of proteins showing that fold-change (Fig. 6). As evident from Fig. 6, a fold-change value of 1.75 was obtained as the inflection point to identify proteins showing significant change in association with Akt1 protein in either G1/S or G2 phase.

**Figure 7:**
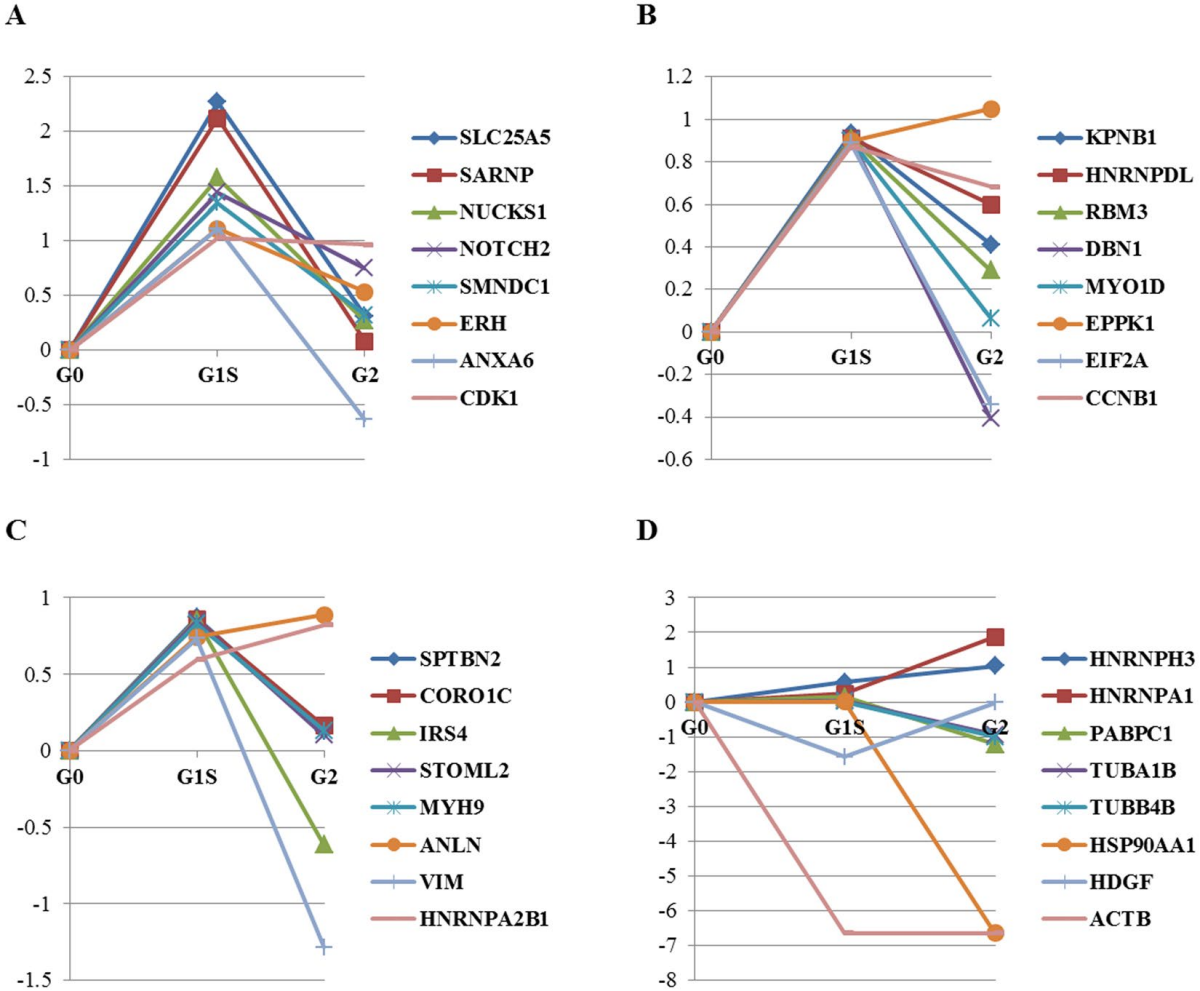
(**A**–**D**) Line graphs depicting cell cycle stage-specific variations in the extent of association with Akt1 protein. The plotted values are the log_2_ values of the obtained dataset.

Hence, using a ratio cut-off of ±1.75 as the threshold of significance, 32 Akt1 interactors could be shortlisted as displaying cell cycle stage-specific variations in the extent of association with Akt1 protein (Fig. 7). Of these, 21 proteins showed increased association with Akt1 in the G1/S phase relative to G0, whereas two proteins (ACTB and HDGF) dissociated from the Akt interactome. In majority of cases showing increased association in G1/S phase, the effect was relatively transient with levels of the association reducing either partially, or to baseline levels, in G2 phase (Fig. 7A–C). The only exceptions to this were CDK1, EPPK1, ANLN, and HNRNPA2B1, where the increase in association seen in the G1/S phase either persisted or further increased in the G2 phase (Fig. 7A–C). Of the two dissociation events noted in the G1/S phase, that of HDGF was specific to this phase as baseline level association was restored in the G2 phase (Fig. 7D). On the other hand, association of ACTB with the Akt1 interactome was restricted to only the G0 phase of the cell cycle (Fig. 7D).

Of the G2-specific events noted, the most significant was the near complete dissociation of HSP90AA1 from its complex with Akt1 (Fig. 7D). In addition, relatively lower levels of dissociation of PABPPC1, TUBA1B, and TUBB4B were also seen. In contrast, we also noted increased association, relative to levels in the G1/S phase, of the ribonucleoproteins HNRNPH3 and HNRNPA1 (Fig. 7D).

**Figure 8:**
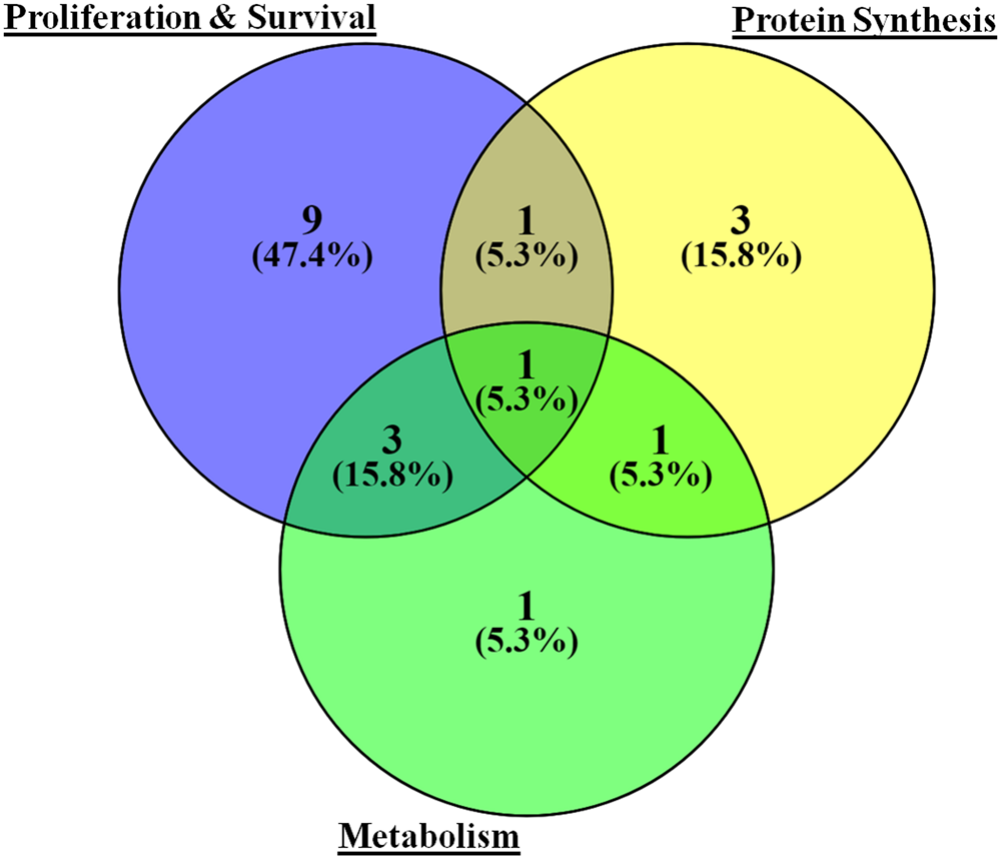
Venn diagram depicting GO distribution of proteins showing significantly altered association with Akt1 protein in G1/S phase of the cell cycle.

A total of 9 out of 23 proteins were implicated in cell proliferation and survival and another 10 represented either protein synthesis or metabolism or both, or in all of the three functional groups (Fig. 8). Table 1 enlists the GO distribution of proteins showing altered association with Akt1 in G1/S phase of the cell cycle.

**Table 1:** GO classification of proteins displaying altered association with Akt1 in G1/S phase.

Gene names	GO classes
ANXA6	Proliferation and Survival
CCNB1	Proliferation and Survival
CDK1	Proliferation and Survival
DBN1	Proliferation and Survival
HDGF	Proliferation and Survival
KPNB1	Proliferation and Survival
MYH9	Proliferation and Survival
NOTCH2	Proliferation and Survival
SMNDC1	Proliferation and Survival
ACTB	Protein Synthesis
RBM3	Protein Synthesis
SARNP	Protein Synthesis
STOML2	Metabolism
SPTBN2	Proliferation and Survival; Protein Synthesis
ERH	Proliferation and Survival; Metabolism
IRS4	Proliferation and Survival; Metabolism
NUCKS1	Proliferation and Survival; Metabolism
EIF2A	Protein Synthesis; Metabolism
SLC25A5	Proliferation and Survival; Protein Synthesis; Metabolism
CORO1C	Others- actin cytoskeleton organization
MYO1D	Others- cellular localization
EPPK1	Others- Cytoskeleton remodelling Keratin filaments
HNRNPDL	Others- RNA processing

Remaining 4 proteins depicted functions related to either RNA splicing, cytoskeleton remodelling, focal adhesion assembly, or cellular localization and movement.

### Functional analysis of Akt1 interactors

**Figure 9:**
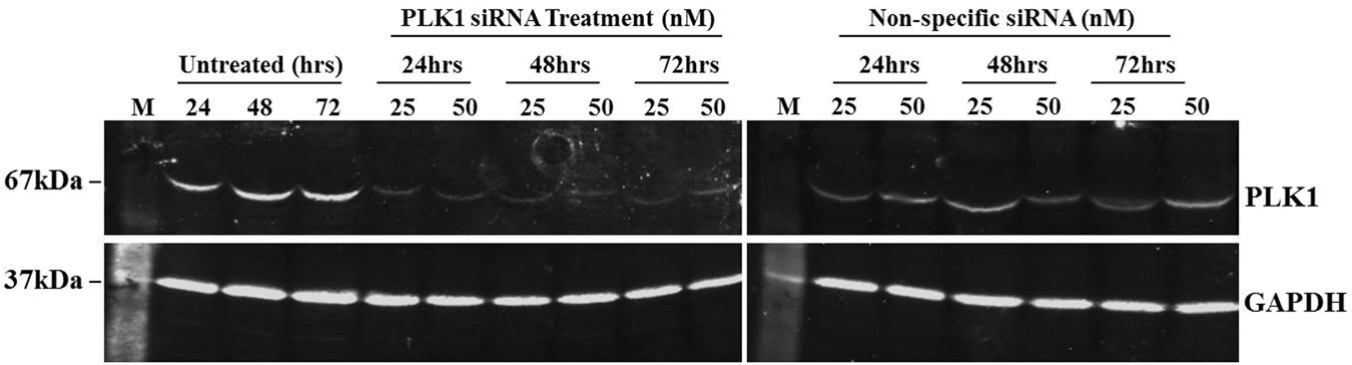
Blot depicting the effect of knockdown of PLK1 expression along with non-specific (NS) siRNA in HEK293 cells. siRNA concentrations tried were 25 nM and 50 nM and the effect of silencing was determined at 24 hours, 48 hours and 72 hours, respectively. Here, M represents the protein marker. GAPDH was probed as loading control.

Given the role of Akt1 in regulating G1/S phase of the cell cycle^6^, we suspected a mediatory role in this process for those interactors that displayed altered association with Akt1 protein in G1/S phase. Here, we particularly focused on those interacting proteins whose association with Akt1 increased as cells transited from the G0 to the G1/S checkpoint. The potential significance of these associations was evaluated through siRNA-mediated silencing of expression of the genes coding for each of these proteins. For this we employed SMARTpool ON-TARGET plus siRNAs to target all of the 23 proteins identified in Table 1. Dosage of siRNA and time required for maximal silencing were determined against PLK1 at 25 nM and 50 nM siRNA concentrations at 24, 48 and 72 hours after transfection (Fig. 9). Maximum effect of the siRNA knockdown was obtained around 48 hours post transfection, which persisted until 72 hours. Therefore, we decided to analyze the effect of siRNA knockdown on expression of target proteins at 48 hours post transfection.

We observed that 25 nM of PLK1 siRNA yielded 70–80% knockdown and the effect was retained up to 72 hours of transfection. In this case also we observed the results to fall in line with PLK1 knockdown. Therefore, a working concentration of 25 nM was used for each target specific siRNA pool for all subsequent transfections. Propidium Iodide was used to stain the DNA content followed by sample acquisition using FACS canto. Untreated, unstained HEK293 as well as stained cells were used to calibrate the desired FACS parameters.

**Figure 10:**
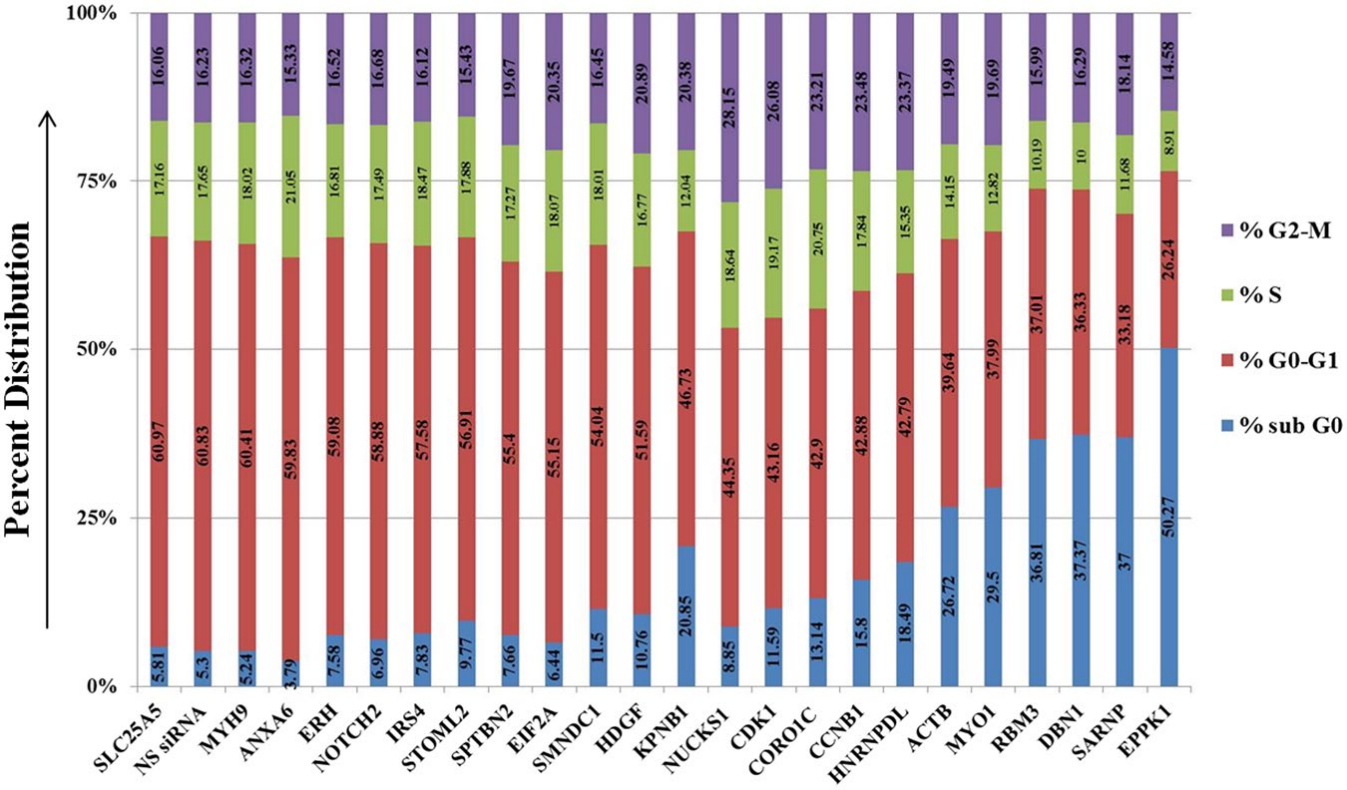
Bar-graph depicting percent distribution of cells in different cell cycle phases after gene knockdown using target-specific siRNAs against the perturbed proteins. Cell cycle phase specific distribution consequent to silencing of PLK1 and Akt1 genes was also determined and revealed a significant increase in G2 and G0 populations, respectively.

Each transfection experiment included PLK1 as the positive control for cell cycle analysis; a non-specific (NS) siRNA was used as the negative control. Compared to untreated cells, PLK1 silencing presented significant increase in G2/M population (52.91%). In line with this, G0/G1 population was depleted by almost 51.43% depicting G2/M arrest of the cell cycle. The effect of the siRNA knockdown on percent population of cells in sub-G0, G0/G1, S and G2/M phase for the targeted genes along with NS siRNA control is provided in Fig. 10.

Interestingly, silencing of expression of several of the target proteins led to a significant reduction in cell viability as evidenced from increase in the sub-G0 population of cells. By taking a cut off of >3-fold over the value obtained with NS siRNA, we identified eight Akt1-interactor proteins to play a prominent role in cell survival. These were KPNB1, HNRNPDL, ACTB, MYO1, RBM3, DBN1, SARNP and EPPK1, with suppression of the latter yielding over 50% cell death. EPPK1, or Epiplakin 1, is a member of the plakin family of proteins. Although plakin proteins play a role in organization of the cytoskeletal architecture, not much is known about the function of EPPK1. It is however expressed in several cancer cells and has been proposed as a marker for detecting pancreatic progenitor cells in developing and regenerating pancreas^18^. Our present finding, however, describes EPPK1 as an important component of the Akt1 interactome, particularly during transition of cells from G0/G1 to the G1/S checkpoint.

Of the remaining seven interactors, Karyopherin Beta 1 (KPNB1) is a nucleocytoplasmic transport protein that mediates nuclear import of histones. Interestingly this protein is overexpressed in some cancers, and has been shown to play an important role both in tumor cell survival and tumorigenicity^19,20^. Further, elevated KPNB1 expression is associated with deregulated E2F/Rb activities^21^, thereby directly implicating a role for this protein in regulation of the cell cycle. The Heterogeneous Nuclear Ribonucleoprotein D like (HNRNPDL) is a member of the HnRNP family and functions primarily as a transcriptional regulator. Not much is, however, known either about its interaction with Akt1, or its role in cell survival. Our results here, therefore, present interesting new information on this protein that is worthy of further enquiry. Beta-actin (ACTB) is a non-muscle cytoskeletal actin involved in cell motility, structure and integrity. Interestingly, this protein has been identified as a downstream substrate of Akt signalling and specifically controls cell growth and migration^22^. Our present finding that the interaction between Akt1 and ACTB is restricted to the interphase, with the complex dissociating during the mitotic phase, could have interesting mechanistic implications.

MYO1 is a member of the nonconventional myosin family that function as actin-based molecular motors^23^. No information is presently available on possible interactions between MYO1 and Akt1. RBM3, the cold-inducible mRNA binding protein, regulates global protein synthesis by enhancing phosphorylation of translation initiation factors on the one hand, and promoting active polysome formation on the other. Interestingly, RBM3 is also known to influence Akt activity by regulating its phosphorylation at Ser473^24^. Thus, the RBM3-Akt1 interaction identified here may serve as at least one of the signalling axes that controls growth during the cell division cycle.

Drebrin 1 (DBN1) plays a critical role in progranulin-mediated activation of the Akt pathway and modulates motility, invasion, and anchorage independent growth of cells^25^. On the other hand, SARNP is a cytokine-regulated protein that directly regulates cell cycle progression^26^. In the absence of any prior information on regulatory interactions between SARNP and Akt, the mechanistic implications of our present results remain to be clarified. Nonetheless, our collective findings reveal that the interphase-specific Akt1 interactome includes proteins that have been implicated in regulating cell survival, cell growth, and progression through the cell cycle. It is not unreasonable to speculate then that it is such interactions that mediate, at least in part, the anti-apoptotic and mitogenic activities of Akt1.

### Effect of target protein depletion on cell PDTs

We next probed the effects of RNAi-mediated suppression of the G1/S-specific proteins of Akt1 interactome on cell PDT and on residence time (RT) in the individual cell cycle phases. Here we again particularly focused on the G1 phase given that mitotic signalling from Akt1 involves transiting cells from G0 and pushing them over the G1/S checkpoint. We specifically scored for proteins where silencing affected RT of the G1 phase by more than 3 hours relative to the control value, and the results obtained are presented in Table 2. As is evident, suppression of several of the targeted proteins yielded a marked effect that involved either a decrease, or an increase, in G1 RT. The group yielding the former effect included CORO1C, MYO1, RBM3, EPPK1, and NUCKS1. In each of these cases, protein silencing led to a significant reduction in G1 RT with EPPK1 suppression producing a 50% reduction in the RT of this phase (Table 2). Interestingly, with the exception of CORO1C, reduction in G1 RT also led to a corresponding reduction in cell PDTs. Here again the effect of EPPK1 suppression was most profound, causing a near 50% reduction in PDT relative to the control value (Table 2). In case of CORO1C, the reduction in RT of G1 was compensated by corresponding increases in those of S and G2, with only a marginal net effect on PDT (Table 2). Thus these latter results support that at least some of the proteins that associate with the Akt1 interactome in a G1/S-specific manner are likely involved in enforcing a regulatory control over the mitogenic effects of Akt1.

**Table 2:** RT for the silenced genes in different cell cycle phases and their cumulative PDT were determined in hours. PLK1 and Akt1 were employed as positive controls for cell cycle arrest. NS siRNA treated sample represented the negative control.

Samples	RT G1	RT S	RT G2	PDT (total of RT)
NS siRNA(Control)	16.82	4.66	5.01	26.49
EIF2A	24.81	8.13	9.15	42.09
ERH	22.54	6.41	6.3	35.25
IRS4	21.55	6.91	6.03	34.49
MYH9	21.48	6.41	5.8	33.69
SPTBN2	19.67	6.13	6.98	32.79
KPNB1	18.61	4.8	8.12	31.52
ACTB	16.92	6.04	8.32	31.29
SARNP	16.09	5.66	8.8	30.55
DBN1	17.61	4.85	7.9	30.36
NOTCH2	19.21	5.71	5.44	30.35
CDK1	14.42	6.41	8.71	29.54
HNRNPDL	15.37	5.51	8.39	29.27
ANXA6	17.91	6.3	4.59	28.81
SMNDC1	17.49	5.83	5.32	28.64
CCNB1	14.51	6.03	7.95	28.49
STOML2	17.86	5.61	4.84	28.32
SLC25A5	17.92	5.04	4.72	27.68
HDGF	15.38	5	6.23	26.61
CORO1C	12.76	6.17	6.91	25.84
NUCKS1	11.36	4.77	7.21	23.34
RBM3	12.88	3.55	5.56	21.99
MYO1	11.61	3.91	6.01	21.53
EPPK1	7.66	2.6	4.25	14.51

RNAi-mediated suppression of levels of six other proteins resulted in a significant increase in the RT of G1. These proteins were ERH, MYH9, NOTCH2, EIF2A, IRS4, and SPTBN2. Importantly, in all of these cases, increase in G1 RT also resulted in a comparable increase in PDT (Table 2). Based on these results it is possible to interpret that these six proteins play an active role in facilitating progression of cells through G1 phase of the cycle. The cumulative results in Table 2, therefore reveal that the G1/S-specific components of the Akt1 interactome collectively play a role in regulating the rate of progression of cells through the G1/S phase of the cycle. The net rate achieved appears to represent a balance between the effects of those proteins that impose a check on this process (CORO1C, MYO1, RBM3, EPPK1, and NUCKS1) versus the effects of those that play a facilitating role (ERH, MYH9, NOTCH2, EIF2A, IRS4, and SPTBN2). While it remains to be proved it is, nonetheless, tempting to speculate that these counter-regulatory effects are necessary to ensure coordinating of cell cycle with cell growth.

Finally, in addition to the above effects, we noted that suppression of four additional proteins also resulted in a significant increase in cell PDT. These proteins were ACTB, DBN1, KPNB1, and SARNP (Table 2). In all of these cases however, increased PDT did not result from any cell cycle stage-specific effect. Rather it derived from marginal influences on all of the individual phases (Table 2).

## Discussion

The serine/threonine kinase Akt/PKB is a central signalling node in all eukaryotic cells and represents the most important kinase at the core of human physiology and disease^4^. It functions downstream of growth factors, oncogenes and cell stress and is best known for promoting cell survival and growth^6,7^. Akt is activated by phosphorylation at Thr308 and Ser473 and this activation also stimulates cell proliferation through multiple downstream targets that impinge on cell-cycle regulation^7,27^. Importantly, by controlling both metabolism and protein synthesis in parallel, Akt ensures that cell growth is coordinated with the cell cycle, thereby inducing cell proliferation^28,29^. To better understand the mechanisms by which it promotes proliferation, we initiated the present study to examine the protein interaction environment of Akt1. The particular emphasis here was to monitor the changes in this environment that occur as cells progress through the cycle. Our hypothesis here was that dynamic modulations in the Akt1 interactome might be causally linked to pathways that drive cells through the cycle.

Affinity purification combined with protein quantitation strategies like SILAC offer a smart handle to study protein-protein interactions. In the present study, cells labelled with different SILAC labels were mixed prior to affinity purification to target the interacting protein partners of Akt1 protein. Although, the employed strategy is very efficient, it is unable to differentiate the stable/strong interactors from transient/weaker ones^30,31^. We attempted to demonstrate a few of the observed interactions to be real through Co-IP followed by reverse Co-IP experiments. Limitation of such an approach is that it requires a lot of protein sample for detection of endogenous expression of proteins and there is high probability of not detecting transient or weak interactions due to limited sensitivity of western blotting. Other techniques like FRET or proximity ligation assay which are highly sensitive, rapid and require fewer number of cells are better available options to validate true protein interactions^32,33^.

An important highlight of our study was the high confidence delineation of the Akt1 interactome, which included the identification of several novel interacting partners. Of particular interest was the fact that over half of the 213 interacting partners identified were derived from GO classes related to processes governing cell growth and the cell cycle. For instance, 96 of these proteins could be characterized under the broad functional class of cell proliferation and survival. Notable examples of these-which are also known Akt interactors-include CALM1, CDC37, GNB1, HSP90AA1, HSPA5, IQGAP1, MAPT, NPM1, PRKDC, TUBB, VCP and YWHAE. Of these VCP, or p97, is particularly interesting since it is associated with a wide range of ATP-dependent cellular processes such as ubiquitin-mediated proteolysis, DNA repair, membrane fusion and dynamics of subcellular compartments, gene expression, and cell growth^34^. It is an essential target for Akt signalling^34,35^. Further, degradation of cyclin E and the G1-cyclin-dependent-kinase inhibitor Far1, which regulates cell cycle G1/S transition is also mediated by VCP/p97^36^.

Another 38 of the interactome proteins could be implicated in protein synthesis, the most notable of these being the eukaryotic translation initiation factor 2A (EIF2A). This protein plays an important role in early steps of protein synthesis and functions by directing the binding of methionyl-tRNA to 40S ribosomal subunits^37,38^. In addition to the interactome components involved in regulation of protein synthesis, another 29 of the Akt1 interactors could be classified as those regulating metabolic functions. These findings are significant given that protein synthesis and metabolism constitute the two cellular functions that contribute towards cell growth. As noted earlier, coordination of cell growth with the G1 and S phases of the cell cycle is critical to ensure productive doubling of the cell. Collectively, therefore, these findings underscore the central role that Akt1 plays in regulating cell proliferation. Importantly by delineating the interacting partners of this enzyme and characterizing their functional properties, our results provide the framework for future investigations into how Akt governs mechanisms that guide cell proliferation. This is clearly an important question given the central role that Akt plays in development and progression of most tumors.

Based on our cut-off, we determined that 23 of the interacting proteins displayed altered association with Akt1 at the G1/S checkpoint, relative to that when cells were arrested at the G0 phase. While the majority of these proteins showed increased association two-ACTB and HDGF-were found to dissociate at this stage. ACTB, a known interactor of Akt1^39^, was dissociated from Akt1 in both the G1/S and G2 stages of the cycle. It plays an important role in most cellular processes such as cell migration, cell division, and regulation of gene expression^40^. These functions are attributed to the ability of actin to form filaments that can rapidly assemble and disassemble according to the needs of the cell^39^. Dissociation of HDGF at the G1/S stage was only transient and its association with Akt1 was restored at the G2/M phase. HDGF is a known survival factor and it is phosphorylated at Ser103 during M phase of the cell cycle^41^.

A functional analysis of the proteins, by using siRNA-mediated silencing, revealed that a significant proportion of the G1/S-specific interactors played key roles in contributing either to cell survival and/or the cell cycle. In the context of the former, the most significant effect was obtained with EPPK1 where cell death resulting from its suppression was comparable to that obtained upon Akt1 silencing. This is clearly an interesting finding and the role of EPPK1 in facilitating cell survival bears further investigation. Another notable result from these experiments was that 15 of the 23 G1/S-specific interactors tested were found to play a significant role in regulating PDT with 11 of these directly impacting the G1 phase of the cell cycle. Thus at least some of the components of the Akt1 interactome either coordinate with, or mediate, the mitogenic function of Akt1. Particularly intriguing here was the fact that suppression of expression of six of the members of the latter group (ERH, MYH9, NOTCH2, EIF2A, IRS4, and SPTBN2) resulted in a marked prolongation of the G1 phase. This would suggest that these proteins collectively contribute towards facilitating progression of cells through the G1 phase under basal conditions. Conversely, RNAi-mediated suppression of an additional five members of the group (CORO1C, MYO1, RBM3, EPPK1, and NUCKS1) resulted in a significant shortening in RT of the G1 phase. These latter proteins, therefore, likely exercise a restraining control over progression of cells through the G1 phase. Collectively therefore, these results indicate that the Akt1 interactome is populated by a core subset of proteins that control progression of cells through the cycle. While a subset of these proteins act by facilitating G1 progression, this effect appears to be balanced by another subset of the G1/S-specific proteins that exert a restricting effect on the G1 phase. Presumably it is the net effect of these counteracting forces – and perhaps other such factors-that eventually determines the kinetic features of cell cycle progression and, thereby, the PDT.

Thus, in summary, our study provides the first experimental description of the Akt1 interactome and also maps its alterations as a function of cell cycle progression. While subsequent results highlight some novel aspects of Akt1 function, they also identify several important questions that warrant further scrutiny. For instance, one important query relates to the functional relationship between Akt1 and its G1/S-specific interactors. Does the activity of the latter group depend upon either the enzymatic activity of, or physical association with, Akt1? The alternate possibility that they function in an autonomous manner to regulate the pro-proliferative role of Akt1 also cannot be ruled out. Addressing such queries will clearly shed more light on processes governing cell proliferation and, more specifically, the molecular mechanisms by which Akt executes its influence over this process. Given the prominent role that Akt plays in the development and propagation of most tumors, the need for gaining insights into such issues cannot be underplayed.

## Methods

### Cell Culture Reagents

A glycerol stock of Human Akt1 ORF was obtained as pENTR221 vector from GE Dharmacon (Human Akt1 ORF Clone ID 100067600). Modified destination vector (pcDNA/FRT/TO), containing SH Tag, was a generous gift from Dr. Matthias Gstaiger (Institute of Molecular Systems Biology, ETH Zurich, Zurich, Switzerland). Flp-In TREx HEK293 cells (#R780-07), LR Clonase II enzyme mix (#11791-100), pOG44 vector (#V6005-20), Zeocin (#46-0509), Blasticidin S (#R210-01), Hygromycin B (#10687-010), Tet (#550205) and Dynabeads Protein A (#100.02D) were obtained from Invitrogen. Xtremegene 9 (#06365787001) was acquired from Roche. DMEM (#12430-054) and RPMI 1640 (#22400-089) were obtained from GIBCO. Trypsin (#CC5027.010L) was purchased from Genetics. Normal FBS (#SH30070.03) as well as Tet screened FBS (#SH30070.03T) were procured from Hyclone. Aphidicolin (#A0781-10MG), Nocodazole (#M1404-10MG), Propidium Iodide (#P4170-250 mg) and Lys C protease (#P3428) were obtained from Sigma. SILAC Labels were procured from Cambridge Isotope Laboratories, Inc. Protease inhibitor cocktail (100X) (#78429) and Pierce anti-HA agarose beads (#26182) were obtained from Thermo Scientific. MagStrep ‘Type 2HC’ beads (#2-1612-002) were got from IBA BioTagnology. Bioconcept Labs Pvt Ltd, Gurgaon, India synthesized the HA peptide [YASFKGPNA]. Nitrocellulose membrane (#RPN303E) was purchased from GE Healthcare. Trypsin protease (#4370282) was procured from AB Sciex. Full range rainbow protein molecular weight marker (#RPN800E) was from Amersham. siRNAs, Dharmafect Transfection Reagent (#T-2001-03), RNase Free Water (#B-003000-WB-100), 5X siRNA buffer (#B-002000-UB-100) were gotten from Dharmacon. RNase A (#19101) was from Qiagen.

### Antibodies for Western Blot

Antibodies used in the present study were: anti-HA (#SC-7392; mouse monoclonal, dilution 1:3000); anti-AKT1 (#SC-5298; mouse monoclonal, dilution 1:1000) and anti-GAPDH (#SC-25778; rabbit polyclonal, dilution 1:1000) from Santa Cruz; anti-CDC2 (**#**77055; rabbit polyclonal, dilution 1:1000); anti-CCNB1 (#4138; rabbit polyclonal, dilution 1:1000) from Cell Signalling Technology; anti-BUB3 (#ab133699; rabbit monoclonal, dilution 1:10000); anti-API5 (#ab56392; mouse monoclonal, dilution 1:1000) and anti-SH3PX1 (#EPR14399; rabbit monoclonal, dilution 1:2000) from Abcam; secondary antibodies were procured from Licor Biosciences-Odyssey goat anti-mouse (#926-32210, dilution 1:15000) and Odyssey goat anti-rabbit (#926-32211, dilution 1:15000).

### Generation of stable cell line

In this study, gateway compatible Akt1 entry vector (pENTR221) was used to generate an expression construct by LR recombination reaction in presence of a suitable destination vector (pcDNA/FRT/TO). The destination vector was modified to include a Strep-HA (SH) tag-containing a streptavidin-binding peptide (Strep) and a hemagglutinin (HA) epitope tag. This SH tag was eventually used to selectively pull-out Akt1 and its interacting partners from complex cell lysates. To generate an inducible Akt1 expression cell line, the expression construct was co-transfected with pOG44 recombinase into Flp-In TREx HEK293 cells, stably expressing the Tet repressor. Transfection was facilitated by Xtremegene 9 transfection reagent. Cells expressing Akt1 construct were selected over a period of 15-20 days in hygromycin-B (75 µg/ml) containing RPMI medium, supplemented with 10% Tet screened FBS. These selected cells were later pooled to expand for pulldown experiments.

Tet concentration required for optimal Akt1 expression was determined by inducing Akt1 expression over a range of Tet concentrations (0.1 µg/ml to 5 µg/ml) and protein expression levels of Akt1 were monitored in presence and absence of Tet using anti-Akt1 as well as anti-HA antibody. Tet was supplemented to culture media every 24 hours to sustain steady Akt expression.

### PDT Experiment

PDT for HEK293 cells was monitored in normal versus Akt1-overexpressing cells over duration of 72 hours after initial administration of Tet. For each observation point, a parallel set of 10^4^ normal and Akt1-overexpressing HEK293 cells were seeded in triplicates, in a 96 well plate in 100 µl DMEM media containing 10% serum. Tet was supplemented to culture media 24 hours after seeding and after another incubation interval of 24 hours, cells were harvested as time 0. Hereafter, a set of cells, in parallel culture, was harvested every 24 hours up to 72 hours. PDT was determined by counting the cells at each time point by trypan blue staining method.

### SILAC labelling to differentiate cell cycle phase specific Akt1 interactome

Akt1-overexpressing HEK293 cells were expanded and cultured in SILAC media containing “light” (K0R0), “medium” (K6R6) or “heavy” (K8R10) isotopes of lysine (K) and arginine (R) for at least 5 cell doublings to allow complete label incorporation. At 70% confluency, Tet (1 µg/ml) was supplemented to the media to induce Akt1 expression. K0R0 labelled cells were arrested in G0 phase by overnight serum starvation which is a widely used method to arrest cells in G0 phase^42^. To do this, normal complete culture media was replaced with serum deprived RPMI and cells were maintained in culture for 16–18 hours at 37 °C in 5% CO_2_ atmosphere. K8R10 labelled cells were arrested in G1/S phase by culturing cells overnight in presence of 5 µg/ml of aphidicolin; a reversible inhibitor of eukaryotic nuclear DNA replication^43^. Similarly, for G2 arrest, 400ng/ml nocodazole was supplemented to the culture media of K6R6 labelled cells and the incubation was continued for 16–18 hours before pelleting the cells. Nocodazole interferes with the polymerization of microtubules thereby arresting cells in G2/M phase^44^. The arrested cells were trypsinized and counted separately by trypan blue staining method. Cells arrested in different cell cycle phases were then pelleted by centrifugation at 1500 rpm for 10 minutes. Multiple cell pellets were made for each cell cycle phase and stored at −80 °C, until used.

### SH-tagged Affinity Purification

Same number of Akt1-overexpressing cells arrested in G0, G1/S and G2 phases were mixed in 1:1:1 ratio and lysed on ice for 1 hour in IP buffer (150 mM NaCl; 50 mM Tris-HCl pH7.5; 1% NP-40; 1X protease inhibitor cocktail and 1 mM PMSF). Cell lysate was cleared off debris following centrifugal separation at 10,000 rpm for 15 minutes at 4 °C. Akt1 protein complexes were selectively purified in parallel sets by targeting Strep and HA tag simultaneously, as instructed in manuals of respective kits. Briefly, strep-tagged Akt1 protein complexes were mixed with 100 µl streptactin magnetic beads and kept for 1 hour incubation on a rotary shaker at 4 °C. Any non-specific interactors were washed away during five consecutive wash steps with five bead volumes of streptactin wash buffer. Akt1 protein and its interactors were eventually eluted in two elution steps with 125 µl strep elution buffer per elution (invitrogen). For purification of HA-tagged Akt1 protein complexes, cleared cell lysates were incubated with 50 µl of pre-washed HA agarose beads for 2 hours at 4 °C on a rotary shaker. After 3 quick washes, with ten bead-volumes TBS-T buffer, Akt1 protein complexes were eluted thrice with 50 µl HA peptide (250 µg/ml). The above purification steps were performed for three such biological replicates to purify Akt1 protein and its interacting partners and the Strep and HA eluates for each replicate were pooled, lyophilized and saved at −80 °C until further processing.

### Protein Digestion and Sample preparation for LC-MS/MS

All three biological replicates were processed for protein digestion separately. To the lyophilized sample, 40 µl of 100 mM ammonium bicarbonate was added, vortexed well, followed by addition of 2 µl of denaturant buffer (AB Sciex). The samples were reduced with 4 µl of reducing reagent (AB Sciex) for 1 hour at 60 °C. 2 µl of cysteine blocking reagent was added for 10 minutes at room temperature to block reduced cysteine residues. Protein digestion was initiated by adding 5 µl of 0.1 µg/µl endo-proteinase Lys C. The samples were kept for incubation for 4 hours in a 37 °C water bath. After a short spin, 1 µl of trypsin (1 µg/µl) was supplemented to the samples and continued incubation for another 12–16 hours at 37 °C. Protein digestion was terminated by adding a drop of formic acid (FA).

Acidified samples were lyophilized and subjected to peptide purification using monospin C-18 columns. Prior to use, the C-18 columns were conditioned with methanol and water and further equilibrated with 3% ACN in 0.1% FA. The lyophilized samples were dissolved in 3% ACN in 0.1% FA and loaded on to C-18 columns and allowed to bind for 10 minutes. The samples were passed twice through the columns to ensure complete binding. After 10 stringent washes with 3% ACN in 0.1% FA, the digested peptides were eluted first in 40% ACN followed by two elutions in 60% ACN. Finally, the three eluates were pooled and lyophilized, for each replicate.

The eluted peptides were re-dissolved in 500 µl of 5 mM ammonium formate (pH 2.5) in 30% ACN and gently vortexed. Cation-exchange cartridge was fixed and conditioned before loading the sample. Subsequent to sample loading, the cartridge was washed thrice with 5 mM ammonium formate (1 ml). Peptides were re-eluted twice with 400 µl of 500 mM ammonium formate (pH 2.5) in 30% ACN per elution and the eluates were pooled for each sample replicate and lyophilized.

### Mass Spectrometry Experimental Design and Statistical rationale

#### LC-MS/MS analysis

All samples were analysed by nano-flow liquid chromatography on a nanoflex system (Eksigent Technologies, AB Sciex) coupled to a triple TOF 5600 Mass Spectrometer (AB Sciex; Concord, Canada). Each biological replicate was injected twice as technical replicates. The system was operated using a binary phase gradient, with solvent A (2% ACN in 0.1% FA) and solvent B (98% ACN in 0.1% FA). For optimal sample delivery reproducibility, auto-sampler was operated in full injection mode overfilling the 1 µl loop with 3 µl of sample. For the measurements of each sample injection, peptides were trapped onto cHiPLC trap, 3 µm, Chrom XP C18CL, 120 Å, 0.5 mm × 200 µm (Eksigent) and separated on a cHiPLC column, 3 µm, Chrom XP C18CL, 120 Å, 15 cm × 75 µm (Eksigent) at 300 nl/minute flow rate, using linear gradient: 5–60% solvent B in 80 minutes, 60–90% for 2 minutes. The column was regenerated by washing with 90% solvent B for 6 minutes and re-equilibrated with 5% solvent B for 22 minutes.

The mass spectrometer was coupled to a Nano Spray Ion Source (AB Sciex), controlled by Analyst software (v.1.6). The ion source was equipped with a 10 μm SilicaTip electrospray PicoTip emitter (AB Sciex) and the eluted peptides were monitored by following ion source parameters-IHT = 130°, ISVF = 2100 v, GS1 = 20, curtain gas = 25. The mass spectrometer was operated in information dependent acquisition (IDA) top10 mode and high sensitivity mode with 500 and 200ms accumulation time for the MS1 and MS2 scans respectively, and 12 s dynamic exclusion, resulting in a total duty cycle of ~2.55 s. Mass spectrometer analysis was performed using TOF-MS1 and MS2 survey scans ranging from 350–1250 and 140–1600 m/z, respectively. The Rolling collision energy was automatically controlled by IDA rolling collision energy parameter script. Selection criteria for the parent ion to be fragmented included intensity-where ions had to be greater than 150 cps, mass tolerance of 50mDa, with a charge state of +2 to +5. An auto calibration of spectra was done after acquisition of every 2 samples using dynamic LC-MS and MS/MS acquisitions of 25fmol β-galactosidase.

#### Data Processing and Analysis

MS-MS acquisition of Akt1 and its interacting protein partners was mediated in 3 different cell cycle phases i.e. G0, G1/S and G2 phase. SILAC labelled cells representing each phase were pooled in three separate sets and probed as biological replicates. Acquisition of each biological replicate resulted in 2 wiff and 2 wiff.scan files, representing its technical replicates. Wiff files were again pooled for each biological replicate before searching against UniProtKB/SwissProt Human database (release April 2015) using protein pilot software, version 4.5 (revision no.1656). The reference database consisted of 20,204 protein entries in the specified release. Protein pilot search was based on the paragon algorithm, a part of default statistics of the software. The following settings were used for paragon searches-(a) Species as Homo sapiens, (b) Lys C and Trypsin as enzyme categories for different runs, (c) Maximum missed cleavages = 2, (d) Fixed modifications as SILAC labels-K6R6 and K8R10; cysteine alkylation by methyl methanethiosulphonate (MMTS), (e) Variable modifications as oxidation at methionine, which is a default option in protein pilot, (f) Identification, SILAC (Lys + 6, Arg + 6) and SILAC (Lys + 8, Arg + 10) as sample types, and (g) “Search Effort” parameter “Thorough ID”, which gives us a broad search of various protein modifications, (h) Mass tolerance for precursor and fragment ions were 0.05 and 0.1 Da, respectively. For each biological replicate per SILAC label Lys C and Trypsin digested files were generated. Following parameters were employed to filter the data in order to obtain a confident data set for subsequent analysis-(a) Automatic Bias correction algorithm for heavy to light ratio was applied. It corrects for unequal mixing during the combination of the different labelled samples in one experiment and removes any experimental or systematic bias. The auto bias factor increases the accuracy of quantification by normalizing variations in initial protein quantities^45^, (b) Proteins at 1% Global false discovery rate (FDR) from fit (G-FDR-fit) were retained. The FDR analysis was performed using Proteomics Performance Evaluation Pipeline Software (PSPEP) that is installed with protein pilot software; (c) Minimum protein confidence threshold cutoff was set to 95%; (d) Identification of at least one unique peptide with 95% confidence across all three replicates.

Lists of proteins acquired after Lys C and Trypsin digestion were pooled per biological replicate. Quantitation ratios between medium and heavy SILAC labelled proteins relative to their lighter counterparts were averaged. Subsequently, we got three protein lists per SILAC label-K0R0, K6R6 and K8R10. This was followed by preparation of a union list of the identified proteins; quantitative estimation was curated manually by merging files from three biological replicates for each SILAC condition. Proteins that were identified in all three replicate sets were retained for subsequent analysis and quantitative ratios between medium and heavy labels with respect to light labels for these proteins were averaged. A final combined file was then prepared for K0R0 (G0 phase), K6R6 (G2 phase) and K8R10 (G1/S phase). A protein qualified to be in the final Akt1 interactors list only if it was identified in all 3 replicate sets for any of the cell cycle phase. Proteins that were identified but not quantified in all three replicates were assigned a quantification value of 0.01 (minimum) or 100 (maximum) after intruding into the peptide SILAC ratios.

To prune the list of Akt1 interactors further of any non-specific interactions with affinity matrix or tag *per se*, SH tagged GFP was over-expressed in HEK293 cells. Interacting partners of GFP protein were selectively eluted as described for Akt1 protein complexes. Proteins that overlapped with Akt1 cell cycle phase specific interactome were removed from the Akt1 interactors list as non-specific proteins. This finally gave us a confident dataset for interacting partners for Akt1. A summary of information on the Akt1 interacting proteins and their quantitation estimates is provided in Supplementary Table 3. Dividing them with that of Akt1 ratio in respective phase normalized quantitative ratios for all Akt1 interactors. This gave uniformity to Akt1 as 1 in the G0, G1/S and G2 phases.

### Polyacrylamide Gel Electrophoresis and Western blotting

From the list of identified Akt1 interactors, CDK1, CCNB1, BUB3, API5 and SH3PX1 were randomly selected to validate their association with Akt1 by western blotting. Asynchronized Akt1 expressing cell pellet was lysed in IP buffer and subjected to affinity purification targeting SH tag as described before. Eluates were pooled and diluted in 1X SDS sample buffer and heated for 10 minutes and resolved on 10% SDS-PAGE. Protein bands were transferred onto nitrocellulose membrane and the latter was blocked using 1:1 odyssey blocking buffer: PBS for an hour at room temperature. Next, the membrane was cut to enable proteins of different sizes to be probed with respective primary antibodies to validate along with anti-HA antibody. The primary antibody dilution was made in odyssey buffer: PBST and incubated overnight at 4 °C on a shaker. After thorough washing the blots were exposed to respective IR labelled anti-mouse or anti-rabbit secondary antibodies at a dilution of 1:15000; diluted in odyssey buffer: PBST. The bands were detected using odyssey infrared scanner.

Reverse co-immunoprecipitation was also done using Dynabeads protein A. The previously detected Akt1 interactors were used as bait proteins and Akt1 was probed as their interacting partner using anti-Akt1 antibody. Antibodies against the selected Akt1 interactors were mixed with 50 µl of dynabeads in separate tubes at a concentration of 1:20–1:70; diluted in 200 µl PBST. The bead-antibody mixtures were kept for incubation at room temperature for 10 minutes. Bead-antibody complexes were pelleted using a magnetic separator and re-suspended in 200 µl PBST for washing. Thereafter, 250 µl of lysate was added to each tube and kept for incubation at 4 °C for 2 hours on a rotary shaker. The beads along with respective antibody-antigen complexes were again pelleted and washed thrice with 200 µl PBST, per wash. Then the beads were boiled in 50 µl of 1X SDS sample buffer for 10 minutes and subsequently cooled. Supernatant was loaded onto 10% SDS-PAGE and probed by western blotting. Anti-Akt1 antibody was used to probe for Akt1 in elution of API5, CCNB1, CDK1, BUB3 and SH3PX1.

### GO Classification

GO classes depicting function of each Akt1 interactor were determined from UniProt (http://www.uniprot.org/) and EnrichR databases (http://amp.pharm.mssm.edu/Enrichr/). After extensive manual curation, the identified Akt1 interactors were segregated into 3 major functional classes i.e. proliferation and survival; metabolism; and protein synthesis. The rest were depicted as a common class called ‘others’ which included proteins with functions like transport, hematopoeisis, etc.

### Gene Knockdown using siRNA

#### Standardization

To determine the optimal concentration of siRNA for transfection, 1.2 × 10^5^ HEK293 cells were seeded in a 12-well plate. siRNA against PLK1 was used as a positive control in all knockdown assays. A range of siRNA concentrations from 10 nM to 50 nM was transfected using dharmafect transfection reagent, as per supplier’s protocol. Changes in protein expression levels were monitored by western blotting at 24 hours, 48 hours and 72 hours, respectively (Supplementary Figure 2). Few more targets (SH3PX1, AKT1, CCNB1 and SLC25A5) were knocked down and the effect of knockdown was determined by western blotting. GAPDH was used as a loading control.

#### Knockdown of the target proteins

Proteins showing perturbed association with Akt1 while the cell progressed from G0 to G1/S phase of cell cycle were knocked down individually in HEK293 cells and investigated by FACS. Each siRNA was re-suspended in 1x siRNA buffer to obtain a stock concentration of 20 µM. Transfection was mediated in triplicates 24 hours after cell seeding; along with appropriate positive and negative controls. Each siRNA was diluted to a working concentration of 25 nM in RPMI to make final volume 50 µl and kept at room temperature for 5 minutes incubation. Likewise, transfection reagent was also diluted in RPMI to make a volume of 50 µl and kept for incubation in similar conditions. Both siRNA and transfection reagent were mixed gently to form complexes and continued incubation for another 20 minutes at room temperature. Final volume was made up to 500 µl with media containing 10% serum. These complexes were then gently added onto cells and continued incubation at 37 °C. Cell culture media was replaced with fresh 10% media after 24 hours. Finally, 48 hours after transfection, the transfection efficiency was determined by monitoring siGLO under Nikon Ti inverted microscope.

### FACS acquisition and analysis

At 48 hours post transfection, the cells were briefly centrifuged and the media was aspirated. Cells were stained with 300 µl of propidium iodide solution containing 0.1 mg/ml propidium iodide (Sigma), 3 µl/ml Triton-X 100 (Sigma), 1 mg/ml sodium citrate (Sigma) and 20 µg/ml RNase (Sigma) for 30 minutes at 4 °C. The stained cells were immediately transferred to BD FACS tubes and acquired in BD FACS Canto.

The data obtained was analyzed using FlowJo software. DNA histograms were analyzed to quantitate sub G0, G0/G1, S and G2/M populations. Minor shifts were observed in the G0/G1 and G2/M populations and hence manual gating was done to analyze these populations.

### PDT and RT Calculation

PDT and RT at G1, S and G2 phases of the cell cycle were calculated following siRNA knockdown of the set of proteins showing perturbed association with Akt1 in G1/S phase. PDT for the 23 genes, along with controls, was monitored in HEK293 cells over 72 hour’s duration. Briefly, 10,000 cells were seeded in a 96-well plate, in 100 µl of RPMI media with 10% serum. siRNA transfection was mediated the next day in triplicate for each target protein and was considered as 0 hour time point. The cell counts were determined for untreated HEK293 cells by trypan blue staining method. After 24 hours, cell culture media containing siRNA complexes was replaced with fresh RPMI containing 10% serum. Thereafter, cells were counted for each of the siRNA transfected cells to represent the 24 hour time point. Henceforward, a set of cells, running in parallel culture, were harvested and counted at 48 hours and 72 hours’ time points, respectively.

Once PDT was determined for all the targeted set of perturbed genes, RT (in hours) for each were calculated for G1, S and G2 phase as follows:

$${\rm{RT}}= \% \,{\rm{population}}\,{\rm{of}}\,{\rm{cells}}\,{\rm{in}}\,{\rm{that}}\,{\rm{phase}}\times {\rm{PDT}}/100$$

where; % population of cells were determined by FACS acquisition.

### Data Availability

The mass spectrometry data was deposited as a dataset “Defining the Akt1 interactome and delineating alterations in its composition as a function of cell cycle progression.” to ProteomeXchange via the PRIDE database. It is publicly available via ProteomeXchange with identifier ProteomeXchange: PXD005557. The data set consists of 12 raw files (wiff and wiff.scan) and associated 18 protein pilot files.

## Electronic supplementary material

Dataset 6

Dataset 1

Dataset 2

Dataset 3

Dataset 4

Dataset 5
