# Supplementary material for: Defining the Akt1 interactome and its role in regulating the cell cycle
Source: Sci Rep. 2018 Jan 22;8:1303. doi: 10.1038/s41598-018-19689-0 (PMC5778034; doi:10.1038/s41598-018-19689-0)

**Defining the Akt1 interactome and its role in regulating the cell cycle**

Shweta Duggal^1,3^, Noor Jailkhani^2^, Mukul Kumar Midha^2^, Namita Agrawal^3^, Kanury VS Rao^1^, Ajay Kumar^1,*^

**Legends to Supplementary Tables and Figures**

Supplementary Table 1: Summary of unused scores, total scores, % coverage and number of peptides for interactors of SH-tagged GFP, identified at 1% G-FDR from fit. The listed interactors were obtained subsequent to lys C and trypsin digestion.

Supplementary Table 2: List of interactors identified in both GFP and Akt1 pull-down experiments. These proteins were identified as non-specific background and hence were removed from the Akt1 interactome list.

Supplementary Table 3: Summary of information on Akt1 interactors identified at 1% G-FDR from fit in replicate sets and their quantitative ratios. Unused scores, total scores, % coverage and number of peptides identified at 95% confidence subsequent to lys C and trypsin digestion are compared for each protein identified in respective cell cycle phases - G0 (K0R0), G2 (K6R6) and G2/M (K8R10). Heavy/light or medium/light ratios for proteins labelled with respective SILAC labels are also compared. Here, 'Y' denotes the positive identification of a protein in G0 phase of cell cycle.

Supplementary Table 4: Processed file depicting mean heavy/light or medium/light ratios for all 213 unique and high-confidence interactors of Akt1. SILAC ratios were further normalized with respect to Akt1 to determine differential association between Akt1 and its interacting partners.

Supplementary Table 5: GO classification of Akt1 interacting proteins. Akt1 interacting partners were obtained to segregate into three major functional classes of Akt1 - proliferation and survival; protein synthesis and metabolism. Protein which did not fall into any of these 3 classes were grouped together as a 4^th^ class as ‘Others’.

Supplementary Figure 1: Depiction of the estimated false discovery rate through a representative set of chromatograms for Lys C and Trypsin digested G0 phase (K0R0), G2 phase (K6R6) and G1S phase (K8R10) samples. Panel A and B depict Lys C and trypsin digested samples, respectively.

Supplementary Figure 2: The effect of siRNA knockdown of 4 targets at 48 hours post transfection. GAPDH was probed as a loading control. Lane M: Marker; Lane 1: Normal HEK293 lysate; Lane 2: lysate from siRNA silenced cells.

Supplementary Figure 1


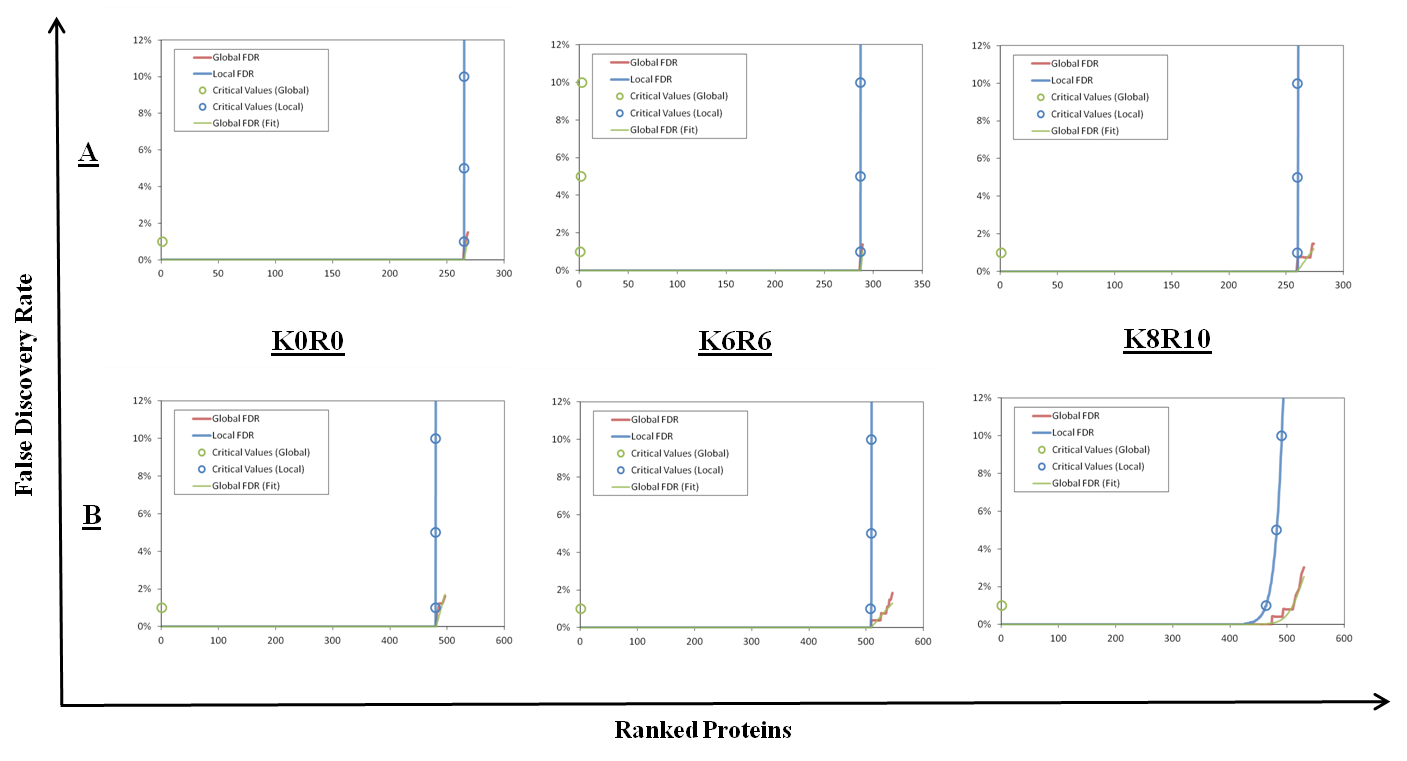


Supplementary Figure 2


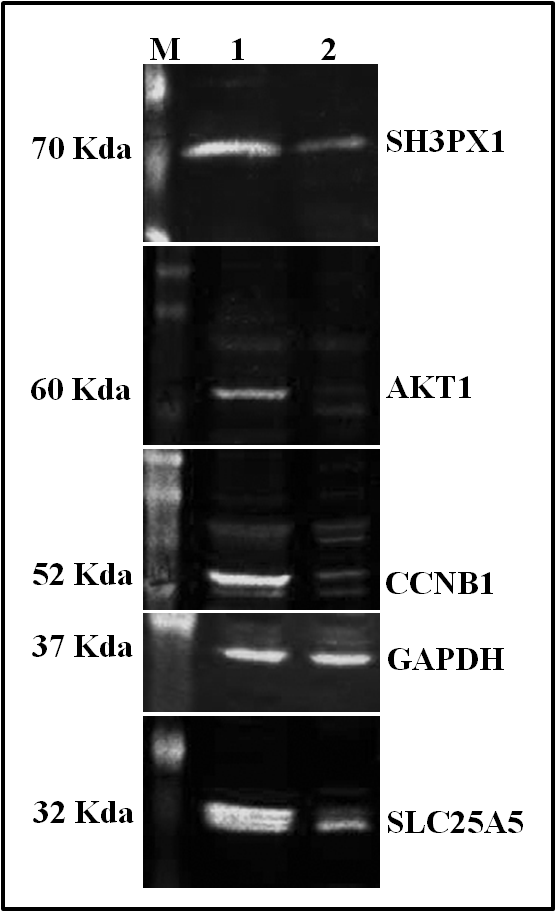

Supplement: Supplementary file 1 — Dataset 6 [file 41598_2018_19689_MOESM1_ESM.doc]
